# Unresectable Moderately Differentiated Gallbladder Adenocarcinoma in an Adolescent: A Case Report

**DOI:** 10.7759/cureus.78308

**Published:** 2025-01-31

**Authors:** Muhammad Sabih, Ishfaq Ahmad Shah, Zarafshan Zakir, Abdul Moez, Zeeshan Uddin

**Affiliations:** 1 General Surgery, Medical Teaching Institution (MTI) Khyber Teaching Hospital, Khyber Medical College, Peshawar, PAK; 2 Oncology, Medical Teaching Institution (MTI) Khyber Teaching Hospital, Khyber Medical College, Peshawar, PAK; 3 Internal Medicine, Medical Teaching Institution (MTI) Khyber Teaching Hospital, Khyber Medical College, Peshawar, PAK; 4 General Surgery, Northwest General Hospital and Research Centre, Peshawar, PAK; 5 Pathology and Laboratory Medicine, Aga Khan University Medical College, Karachi, PAK

**Keywords:** adenocarcinoma, cancer in adolescent, chemotherapy response, gallbladder cancer (gbc), palliation

## Abstract

Gallbladder carcinoma (GBC) is a relatively rare disease of old age with adenocarcinoma being the most prevalent subtype. It is extremely rare in childhood and adolescence, and only a few cases have been reported to date. A 15-year-old boy presented in the outpatient department referred from the periphery with dull progressive right upper quadrant abdominal pain, associated with jaundice, abdominal distension, anorexia and significant weight loss for the past three months. The patient’s medical history was inconclusive. Laboratory evaluations revealed obstructive jaundice. Imaging showed an infiltrative mass at the porta hepatis which upon biopsy demonstrated a moderately differentiated gallbladder adenocarcinoma. Percutaneous transhepatic biliary drainage (PTBD) was done for symptom relief. Chemotherapy with a combination of gemcitabine and carboplatin was opted with palliative intent. After three cycles the patient still showed no response, so the chemotherapy was stopped and only symptomatic treatment was continued.

Urgent provision of medical care upon the development of symptoms, early sonographic diagnosis and timely cholecystectomy along with adjuvant chemotherapy are crucial in significantly prolonging survival and reducing patient suffering. Gene panel testing and chemosensitivity assay further optimize treatment and help in better understanding of disease.

## Introduction

Gallbladder carcinoma (GBC) is the most common malignancy of the biliary tract and the third most common gastrointestinal (GI) tract malignancy [1] with adenocarcinoma being the most common subtype [2]. Gallstones are the single most important predisposing factor for GBC. The risk increases with age and has two peaks, with the first at 50-60 years and the second at 70-80 years of age [3]. The higher incidence among females is likely linked to their hormones and cholesterol cycling [4]. Congenital defects like choledochal cysts and pancreaticobiliary maljunction (PBM) also pose a risk for GBC later in life [5]. Gallbladder cancer is extremely rare in childhood and adolescence; only a few cases have been reported so far without mentioning any cause and our case is an addition to it.

## Case presentation

A 15-year-old boy presented cachectic and icteric in the outpatient department referred from the periphery with progressive right upper quadrant abdominal pain that was gradual in onset, dull and non-radiating, with jaundice, abdominal distension, anorexia, and weight loss for the past three months. The symptoms progressively worsened and during this time his bowel habits were normal. The patient's history and family history were insignificant. His BMI was 16 kg/m^2^.

Laboratory investigations showed normal complete blood count (CBC), serum electrolytes and renal function tests (RFTs). Liver function tests (LFTs) were deranged as shown in Table 1. Serum tumor markers alpha-fetoprotein (AFP) and carbohydrate antigen 19-9 (CA19-9) also came out to be in the normal range.

**Table 1:** Liver function tests (LFTs) showing obstructive pattern of jaundice.

Investigations	Patient Values	Normal Range
Total bilirubin	13.16 mg/dL	0.3-1 mg/dL
Indirect bilirubin	3.86 mg/dL	0.2-0.8 mg/dL
Direct bilirubin	9.303 mg/dL	0.1-0.3 mg/dL
Alanine aminotransferase (ALT)	68.8 U/L	4-36 U/L
Alkaline phosphatase (ALP)	451 U/L	130-340 U/L
International normalised ratio (INR)	1.69	0.8-1.1

**Figure 1:**
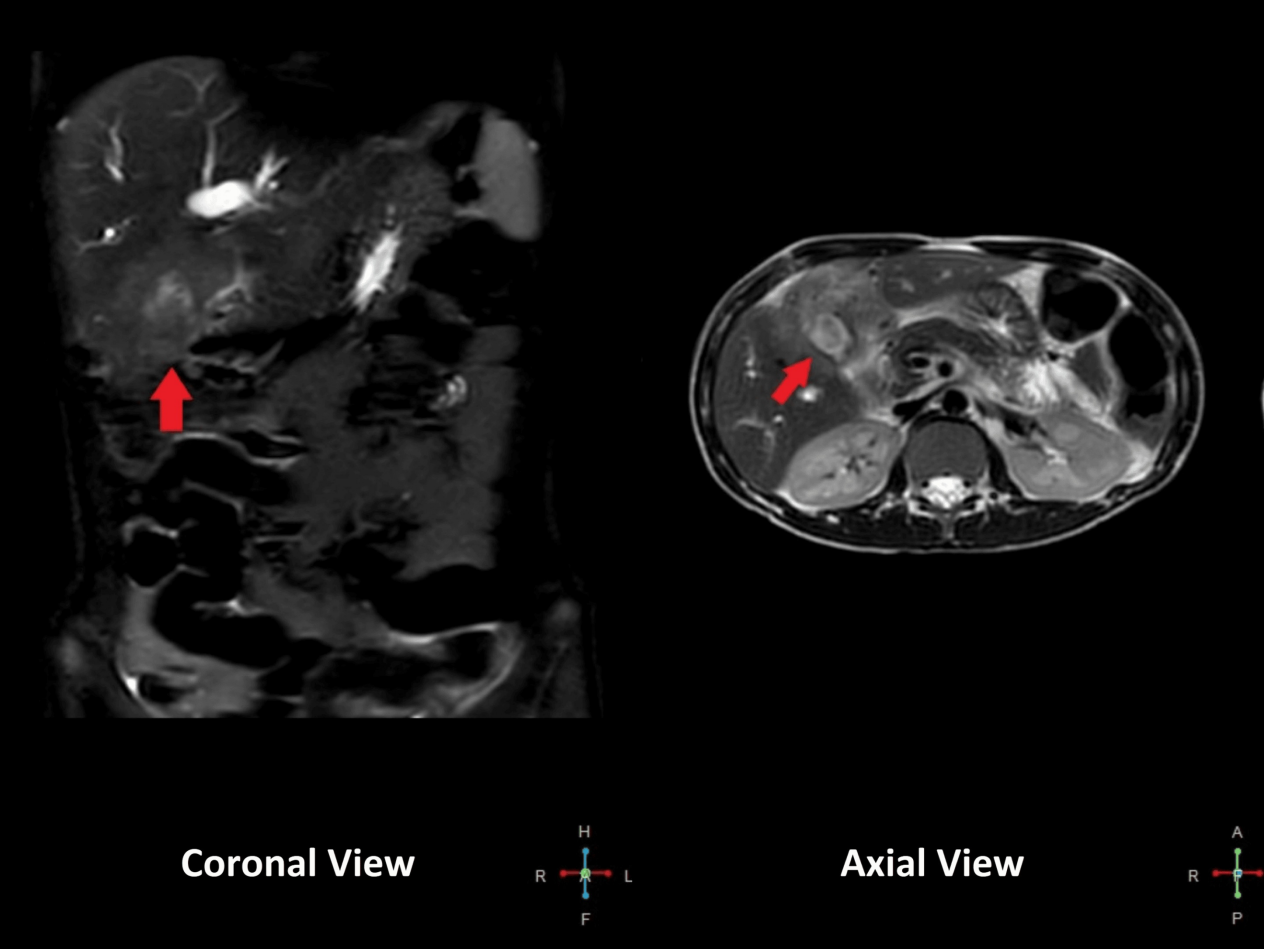
Magnetic resonance cholangiopancreatography (MRCP) showing malignant mass encasing the confluence of right and left hepatic ducts with resultant intrahepatic dilation of bile ducts and porta hepatis extension and encasement of portal vein.

Abdominal ultrasound showed a mass replacing the gall bladder fossa. Computed tomography (CT) showed an infiltrative neoplastic mass at the porta hepatis involving the gallbladder with resultant intrahepatic cholestasis and encasement of the portal vein. There was no evidence of PBM. Magnetic resonance cholangiopancreatography (MRCP) showed a 4.2 x 5.3 x 4cm ill-defined infiltrative mass surrounding the gallbladder and invading the adjacent hepatic parenchyma. Mass is encasing the confluence of right and left hepatic ducts with resultant intrahepatic dilation of bile ducts. At porta hepatis mass is encasing the portal vein without any evidence of intraluminal thrombus. Prominent lymph nodes were seen in the porta hepatis and pancreaticoduodenal region as displayed in Figure 1.

**Figure 2:**
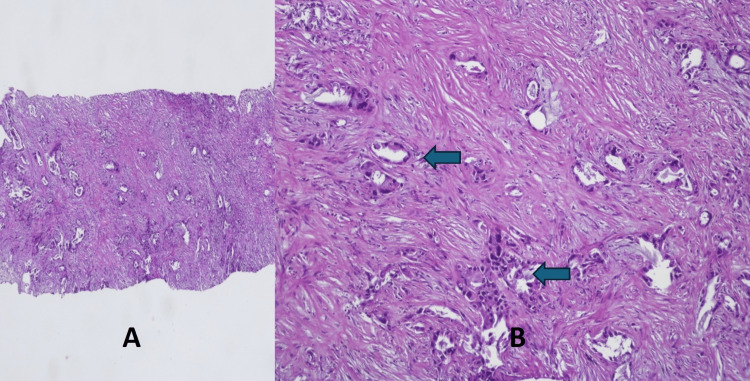
Tru-cut biopsy of the gallbladder mass showing infiltrating glands against a desmoplastic stroma (A). Higher power view (B) exhibiting well to poorly formed neoplastic glands lined by tumor cells with cytological atypia (arrows), consistent with moderately differentiated adenocarcinoma. (Hematoxylin & Eosin, Magnification A: 10X, B: 20X)

An ultrasound-guided Tru-cut biopsy of the mass was performed which showed moderately differentiated gallbladder adenocarcinoma shown in Figure 2. Immunohistochemistry (IHC) stained positive for cytokeratin (CK) 7 and 19.

According to the Union for International Cancer Control (UICC) classification, it was an unresectable stage 4B cancer. Percutaneous transhepatic biliary drainage (PTBD) was done for symptom relief. Three cycles of chemotherapy with a combination of gemcitabine and carboplatin were given with palliative intent. Gene panel testing could not be offered due to unaffordability. Three months later abdominal ultrasound showed progression of the disease with multiple intrahepatic, hypo-echoic lesions in segment V, focal peritoneal thickening in the pelvis, right-sided mild hydronephrosis, hydroureter and moderate to gross ascites. Chemotherapy was stopped and therapeutic ascitic taps were continued along with adequate pain control with oral morphine analogs. The patient was encouraged to use formula milk for better nutrition.

## Discussion

GBC is a common GI tract malignancy with gallstones being the most important and common cause in adults but in children and adolescents, congenital causes (choledochal cysts, PBM and biliary atresia) and infectious causes (chronic typhoid infection of the gallbladder) have been mentioned in the literature. Studies show that mutations involving KRAS, TP53, c-erb-B2 and P16 genes are highly associated with GBC [6]. 

The presenting symptoms of GBC are non-specific including abdominal pain, anorexia, nausea, jaundice and vomiting that are similar to those of other common gastrointestinal tract pathologies. Moreover, the lack of gallbladder serosa allows for early hepatic invasion and metastasis. This leads to late presentation with advanced often inoperable disease at the time of diagnosis. Thus overall prognosis for GBC is poor with a survival rate of less than 20% in five years despite improved diagnostic and therapeutic techniques [7]. 

Surgery is the preferred treatment modality with radical cholecystectomy being the treatment of choice in the early stages of the disease. It involves en-bloc removal of the gallbladder, a wedge of liver tissue from the gallbladder (GB) fossa and all regional lymph nodes around GB fossa. Patients with advanced tumors, metastatic disease or poor health are not candidates for radical resection and thus suitable palliation should be offered [8]. Gemcitabine and cisplatin, alone or in combination are the first-line options as an adjuvant and palliative chemotherapy which can shrink the tumor, relieve symptoms and sometimes allow for its surgical resection [7]. Biliary drainage via stenting or catheter (e.g. PTBD) improves jaundice and the rest is symptomatic management. 

Very few cases of GBC in children are reported till now among which one was a nine-year-old girl who presented early for which she underwent cholecystectomy with T-tube insertion followed by adjuvant chemotherapy. She had no evidence of recurrence in the next 24-month follow-up [9]. In another case, a 15-year-old boy in India presented late with an advanced disease for which supportive treatment was offered and he died three months later [10]. 

These previous reports suggest that early sonographic detection of GBC significantly improves outcomes and thus survival. Advanced genetic studies like next-generation sequencing (NGS), whole exome sequencing (WES), RNA sequencing (RNAseq) and single-cell analysis can identify genetic and epigenetic features of GBC and key molecules for targeted chemo and immunotherapy [11]. This will improve our understanding of the disease process and help optimize our treatment regimens.

## Conclusions

The patient’s late presentation resulted in advanced unresectable disease with no response to palliative chemotherapy. Due to unaffordability, we could not offer gene panel testing which could have optimized our treatment regimen and thus survival. Urgent provision of medical care upon the development of symptoms, early sonographic diagnosis and timely cholecystectomy along with adjuvant chemotherapy are crucial in significantly prolonging survival and reducing patient suffering.
